# Finance’s Social License? Sugar, Farmland and Health

**DOI:** 10.34172/ijhpm.2021.11

**Published:** 2021-03-13

**Authors:** Kiah Smith, Geoffrey Lawrence

**Affiliations:** School of Social Science, The University of Queensland, Brisbane, QLD, Australia.

**Keywords:** Social License, Corporate Accountability, Australia, Financialisation, Investment

## Abstract

**Background:** This paper examines the exclusion of public health from social license narratives within an increasingly financialised food system, through a case study of foreign ownership in the Australian sugar industry. As finance actors such as asset management firms, pension funds, private equity funds, state owned enterprises and sovereign wealth funds engage in speculative farmland investment, commodity futures trading, and the conversion of farmland into a financial asset, power within agro-industrial food supply chains becomes increasingly concentrated. This has been associated with increased food prices and more processed food, contributing to obesogenic diets, hunger, and poorer health outcomes for many. The potential for negative social and environmental impacts has prompted awareness of the need for financial actors to demonstrate sustainability, responsibility and accountability in their farmland investments.

**Methods:** This paper uses thematic analysis of qualitative interviews and key documents to assess four recent acquisitions of sugarcane land in North Queensland. We consider how companies’ efforts to establish or maintain a social license to operate (SLO) intersect with their capital accumulation strategies.

**Results:** Our findings demonstrate that the link between the commodification of ‘unhealthy’ food inputs (such as sugar) and financialisation remains outside the purview of financiers. Instead, agribusiness firms use narratives centred on biofuels investment and energy/food security to justify their legitimacy in the sugar sector. We organise our findings according to two ‘narratives:’ constructing trust and credibility through ethical compliance; and, biofuels expansion as a legitimate response to climate change. The concept of social license is much stronger in the second narrative, but health is largely missing.

**Conclusion:** The ‘distancing’ between responsibility and health outcomes highlights the limits to principles of responsible financial investment, and to the legitimacy of finance to claim an *SLO* – the ongoing approval and acceptance by society to conduct its activities.

## Background

Australia is the world’s second largest exporter of raw sugar, producing for global supply chains characterised by increasing corporate concentration and financialisation. Sugarcane production is of particular interest for financial investors based on its capacity to generate profits in multiple ways via real asset trading (ie, sugar), capital appreciation (land, infrastructure), futures speculation, and material uses (food, fuel, feed). Sugar is also an important ‘flexcrop’ (defined as a crop or commodity whose uses can be flexibly interchanged) associated with the shift to renewable energy.^1^However, as financial actors such as agribusiness firms, asset management companies, pension funds, private equity funds, state-owned enterprises, development banks and sovereign wealth funds increasingly engage in speculative farmland investment,^2,3^ power within agro-industrial food supply chains becomes increasingly concentrated. This has been associated with increased food prices, more processed food and less diversity (in terms of foods and agricultural ecologies), in turn contributing to obesogenic diets, hunger, and ultimately, to poorer health outcomes for many.^4^ In North Queensland, sugar is publicly debated as both friend (valuable global commodity, new energy source, local employer and environmental innovator) and foe (polluter of the Great Barrier Reef, historical exploiter of labour, and a key contributor to poor nutrition and the obesity epidemic). Additionally, recent debates about a ‘sugar tax,’^5^ a banking Royal Commission^6^ and a pending Senate review of foreign investment^7^ further indicate widening public concerns about financial power, regulation and shifting expectations of corporate governance and accountability in the sector. How to ensure socially- and environmentally-responsible financial investment considering the complex ‘web of investment’^8^ has emerged as an important tension – one requiring further research.

This paper presents an analysis of agribusiness’ investment in the sugar industry in Australia by exploring social license ‘narratives’ and how these intersect with health. Since 2010, there have been four major acquisitions of sugarcane-producing land in North Queensland by foreign companies Proterra, Wilmar, Mitr Phol and COFCO (China National Cereals, Oils and Foodstuffs Corporation). Each of these sugar producers is a major financial player in food production, processing, and trade with ties to the top four global commodity traders and agri-financiers, the so-called ABCDs^[1]^. Drawing upon political economy concepts of financialisation and flexcrops, the case studies presented here are used to build a deeper understanding of finance’s ‘social license to operate’ (SLO). This emergent concept refers to “the extent to which a corporation is constrained to meet societal expectations and avoid activities that societies (or influential elements within them) deem unacceptable, whether or not those expectations are embodied in law” (p. 346).^9^ The concept goes beyond corporate social responsibility (CSR), corporate social accountability (CSA) or environment, social and governance (ESG) criteria, which focus mainly on “voluntary approach[es] that a business enterprise takes to meet or exceed stakeholder expectations by integrating social, ethical, and environmental concerns together with the usual measures of revenue, profit, and legal obligation”(p. 3).^10^ We ask: How does foreign capital investment into sugar intersect with the industry’s need to generate an SLO built on a “broad, ongoing approval and acceptance of society to conduct its activities?”^9^ (p. 346). Specifically, do agribusiness financiers give consideration to the health implications of sugar in their efforts to maintain an SLO? Findings draw from qualitative analysis of interviews and document analysis to reveal how agribusinesses seek to legitimate their investments in sugar supply chains.

###  Agribusiness Expansion, Financialisation and Responsible Investment 

In line with agri-food financialisation – broadly defined as the “ways in which the world of finance has come to exert more influence over agriculture and food” (p. 2)^11^ – agribusiness investment across assets of farmland, agricultural infrastructure, processing, trading and retail, and the vertical and horizontal supply chain integration that this represents, has replaced agricultural land as the main focus for current and future financial investment.^12^ For financiers and investors, agribusiness investment is driven by the thematic of cheap, underutilised and undercapitalised land within a context of increasing demand for processed foods. These food industries require a steady source of globally-sourced inputs (sugar, oils, starch), guaranteed access to proteins (milk, animal fats), and a secure pathway to market. A further driver relates to the benefits that speculative investment in farmland, food supply chains, agricultural commodity futures, and other financial assets, have for investors.^13,14^ Through assetisation processes, farmland – which was previously viewed as a risky or poor investment – is converted into a new asset class in ways that enable diverse financial actors to benefit from increased land values and growing demand for food security.^15-17^ This has occurred alongside an ‘agrofuel boom’ – the channelling of food crops into fuel production, or flexcrops – which has underpinned major political-economic restructuring of food prices, land use, energy generation and patterns of financial investment.^18^ Both have contributed to growth in so-called large-scale land acquisitions (LSLAs) by new institutional finance actors. LSLAs have been described as the new resource ‘enclosures’ driven by multiple food, energy, climate and financial crises,^19^ while flexcrops respond to these crises by presenting an ‘ecological fix.’^20^ While institutional finance has increased, transnational agribusiness corporations remain central to this analysis considering that most global farmland and agricultural value chain investment is done by private or public corporations.^11^

The negative impacts of financialisation, and LSLAs especially, have been widely studied^[2]^. Research demonstrates that speculation in agricultural assets (farmland, food and fuel commodities) alongside agri-financial assets (derivatives, futures indexes, equity buyouts) by financial entities – has contributed to food price volatility and the driving up of land and food prices.^13,21,22^ In terms of health, financialisation scholars have argued that processed foods “stimulate overeating and thereby maximise dividends for stockowners”^23^(p. 761), increasing the profitability of inputs to food manufacturing and making this sector one of the most powerful in agri-food supply chains. Numerous reports have also investigated human rights abuses by agribusiness corporations pursuing LSLAs for the production of inputs such as soy, palm oil and sugar.^24,25^ These studies have identified lack of free prior and informed consent, forcible displacement, and violence against local communities which reject corporate ownership over local peoples’ land titles.^26,27^ It has been argued that LSLAs become ‘land grabs’ when they violate human rights, fail to gain consent, disregard social and environmental impact assessments, avoid transparent contracts and do not entail democratic planning, independent oversight or meaningful participation.^19,27,28^ Overall, there is an absence of transparency and accountability in LSLAs by financial actors.^29^

As the potential for harm has been widely acknowledged, so has the need for stronger international mechanisms to govern agricultural investments. National regulations are inadequate for regulating foreign investment,^26^ and international investment law is not designed to influence how investment should contribute to social and environmental outcomes.^30^ While agribusiness has responded to some sustainability issues in their supply chains – such as biodiversity, food security, water use, ethical sourcing, and local livelihoods – there is little evidence to suggest that public health concerns related to consumption of agribusiness’ products have made their way onto the corporate accountability agenda at all. For example, an assessment of 41 corporate sustainability projects drawn from 500 of the world’s largest multinational corporations in food and agricultural supply chains found that none of the projects’ key objectives related specifically (or even, non-specifically) to health. Instead, they focused on long-term sustainability of supply chains (49%), risks to community including food security and economic livelihoods (27%), and voluntary standards compliance (12%).^31^

This last finding mirrors the recent emergence of numerous voluntary, non-binding ‘responsible investment initiatives’ namely: Principles for Responsible Agricultural Investment (PRAI); Principles for Responsible Investment in Farmland (the *Farmland Principles*, or PRI); FAO-led Voluntary Guidelines on the Responsible Governance of Tenure of Land, Fisheries and Forests in the Context of National Food Security (the *Voluntary Guidelines*); and, Principles for Responsible Investment in Agriculture and Food Systems (PRIAFS).^32^ These are all highly contested. Only the PRAI and PRI make specific mention of private financial actors – having had substantial input from financial actors such as the World Bank and institutional investors. The *Farmland Principles*, for example, currently have 16 large institutional investor signatories. The *Voluntary Guidelines* have been criticised for a lack of civil society engagement and failing to protect human rights.^33^ Only the civil society-driven PRIAFS refers to the contribution of investment to food security and *nutrition*, safe and *healthy *agriculture and food systems, and social impacts of investment on *public health and safety *(p. 11, 16).^34^ Although the legitimacy of these voluntary approaches is increasing, they lack accountability mechanisms,^30^ are difficult to enforce, have low participation rates and are confusing due to multiple competing initiatives.^32^ Power and legal rights are mostly granted to private companies who prioritise commercial over public interests, including public health and the environment.^26,35^ This critique has been voiced by public health researchers who have called for greater attention to how investment might redirect the ‘nutrition transition’ away from the overconsumption of processed (ie, unhealthy, sugary) foods.^36^ Literature on how agribusinesses or investors consider their responsibilities regarding health is scarce, although some research on the link between investment, risk and public health is emerging in the context of coronavirus disease 2019 (COVID-19)^[3]^.

###  Social Licence: Beyond Voluntary Accountability

Responsible investment principles and guidelines make up only part of financiers’ social accountability. In their study of biofuel investment in the global South, van Gelder et al^37^ found that “few financiers have criteria in place in order to ensure sustainable investing practices, and those who do tend to have policies of limited quality” (p. 134). In Brazil, for example, institutional investment in sugarcane companies made up two thirds of total investment into biofuels between 2000 and 2009, second only behind palm oil investment. A systematic evaluation of these companies’ responsible financing policies revealed no evidence that investors engaged in screening companies based on responsibility criteria before purchase or thereafter.

In light of this, we contend that ‘SLO’ provides further insight into finance’s legitimacy, beyond voluntary accountability. The concept, originating in business and sustainability fields, has mostly been applied to mining although scholarship has been relatively limited.^38^ SLO goes beyond formal accountability initiatives or state regulations to govern financialisation, to also encompass indicators of broader social acceptability of the activities and outcomes of increasingly financialised industries. Defined by Nelson,^39^ SLO is a “means to earn accountability, credibility, flexibility, and capacity for both stakeholders and industry” (p. 161). Social license represents both a tangible form of risk minimization and profit maximization by companies, and an intangible ‘vision’ that needs flexibility and acceptance in order to accommodate different social paradigms, cultures and social norms as they evolve.^39^ Its three components are *legitimacy, credibility *and * trust*.^9^ The concept is a useful one, considering that community acceptance of financiers’ interests inevitably ‘collide’ with the values, social practices and social/political institutions of the local population or culture.^38^ Nutrition and healthy eating are key examples of social interests that intersect with agribusiness’ broader role in food systems.

Sugar presents a particular paradox for theorising finance’s social license, especially around public health where no voluntary mechanisms beyond labour standards (in production) exist. The multiple uses of sugar (food, fuel and feed)^20^ can be interchanged when it suits, based on narratives that can also be used selectively by agribusinesses, as Borras et al^1^ explain:

*“When sugarcane prices are high, sell sugarcane. When ethanol prices are high, sell ethanol … Meanwhile, build a storyline about this projected scenario to jump-start business undertakings, eg, to raise investments, lure investors, entice governments, persuade affected communities and orchestrate favourable media attention to achieve some of these requirements” *(p. 94).

This new “multiple-ness” of sugar is associated with the changing political economy of diets, public health concerns, and the narrative of climate change prompting socio-political support for renewable energy resources.^1^ But sugar is also a key input for transnational ‘unhealthy’ (ie, processed) food chains and has been directly linked to rising rates of overweight, obesity and chronic disease, including type-2 diabetes.^40,41^ Globally, the large-scale cultivation of sugarcane has driven deforestation and the reduction of biodiversity, human rights and labour abuses, land grabs and ‘depeasantisation.’^42^ SLO has not been widely examined within political economy studies of financialisation or flexing, with studies tending to focus on voluntary CSA initiatives. We ask: What are the ‘social license’ narratives being used by agribusiness investors as they seek to legitimate their role in Australia’s sugar sector, particularly around health? How do these agribusinesses consolidate their financial power, and who benefits and who is disadvantaged?

## Key Messages

**Implications for policy makers**

- New firms entering the sugar industry in Australia give limited consideration to the wider health implications of sugar consumption.
- Voluntary principles of ‘responsible financial investment’ are not sufficient to guide firms in the sugar industry to operate, and sell their products, in a socially-responsible manner.
- Firms in the sugar industry use narratives about food security and energy/climate change to claim a social license to operate (SLO), however these strategies are primarily motivated by financial risk and profit (rather than public benefit).
- While firms in the sugar industry consider they have an SLO, their activities – and mounting opposition to those activities – demonstrate they are falling short in relation to social/health expectations.
- Based upon the evidence, policy-makers in federal and state governments should develop new, compulsory, regulations relating to the production and sale of sugar and ensure that investors in the sugar industry comply with those regulations.

**Implications for public**

Despite the evolution and application of voluntary frameworks for responsible financial investment, these are largely ignored by agribusiness investors in Australia’s sugar industry. Discussions about health do not inform the industry’s narratives relating to accountability. Instead, firms focus upon growing their supply chains and bioenergy capacity. This means that sugar production does not meet community standards; these firms cannot claim they have a ‘social license to operate’ (SLO). Regulating the conduct of investing companies is crucial to developing a more responsive, accountable, transparent and trustworthy sugar industry in Australia. Public health concerns should play a role in determining these regulations.

## Methods

###  Case Study

Sugar in Australia is an important – yet under-researched – area for the study of financialisation, social license and public health. This is important because Australia has seen (*a*) recent high-profile purchases of land and infrastructure by foreign agribusinesses, (*b*) expanding biofuel/energy production and investment, and (*c*) strong public and political contestation around sugar consumption, including the health and nutritional impacts of sugary foods. The industry has approximately 4050 growers, 4000 sugarcane farms, and 24 mills owned by 9 companies.^43^ While the majority of sugarcane *land* in North Queensland is privately owned, some 90% of the industry overall (ie, crushing capacity) is foreign owned. Wilmar, Proterra, Mitr Phol and COFCO together control over 70% of the region’s agricultural/sugar assets, including farmland, sugar mills and other infrastructure.^44^ Table shows the extent of these investments, and where the finance originated.

**Table:** Details of Case Studies

**Australian Company**	Wilmar Australia (43%)	Racecourse Projects (11%, part of Mackay Sugar)	MSF Sugar (13%)	Top Glory/Tully Sugar (5%)
**Parent Company**	Wilmar International	Proterra/Cargill/Black River	Mitr Phol	COFCO
**Financing Country**	Singapore	US	Thailand	China
**Purchase Year**	2011	2013 to 2015	2011	2013
**Land (ha)**	6600, including 2500 from Sucrogen	>6500	13000^a^	Unknown
**Location of Assets in Australia**	Burdekin, Proserpine	Mackay, Marwood Farm	Maryborough	Tully
**Purchase Price**	A$1.75 billion	A$16 million	A$324 million	A$145 million
**Annual Cane Crushed (‘000t) 2017-2019**	14780	1484	4466	2456
**Annual Sugar Produced (‘000t) 2017-2019**	2120	~191	~607	~300
**Other Interests**	Sweeteners, (palm) oil, processing, bioethanol, commodity trading	Raw milk, beef, processing, mining	Processing, sweeteners, ethanol	Grains, soy, palm oil, cotton, sugar, commodity trading, real estate

Abbreviation: COFCO, China National Cereals, Oils and Foodstuffs Corporation. ^a^ In the time since this research was conducted, Mitr Phol has divested approximately 5409 ha of this land to a multinational farmland investment fund.

Each of these four agribusinesses operates as an Australian subsidiary funded by transnational corporate agribusiness firms. These firms engage in productive and financial activities on a global scale, reflecting the processes of financialisation outlined previously. Their Australian operations include land ownership, sugarcane and bagasse production, bioethanol and energy co-generation, sugar milling and processing, infrastructure such as storage and equipment, transport and logistics including port and rail infrastructure, sugar marketing and commodity trading. As will be later discussed, all of these firms also own substantial assets in sugar supply chains globally, including food manufacturing firms and retail outlets. They are deeply embedded in transnational circuits of finance with varying track records on social and environmental performance. These are important factors contributing to the social legitimation of their investment strategies, and underscore myriad tensions associated with the multi-scalar dimensions of firm ownership and operation that have been documented elsewhere.^45,46^

###  Data Collection and Analysis

We present findings from qualitative thematic analysis of eight detailed, semi-structured, interviews conducted between 2016 and 2019 with corporate elites (agribusiness managers, financial intermediaries, investors) specifically engaged in facilitating the flow of foreign finance into agribusiness expansion into Queensland sugarcane land, processing and marketing. Interviews asked about the (*a*) history of the firm, financing structure and transnational relationships; (*b*) drivers, logics and impacts of recent investments in farmland and/or agribusiness supply chains in Australia, including into ‘flexcrops’ where appropriate; and (*c*) corporate goals, activities and challenges associated with corporate accountability, SLO and ESG performance metrics. Written informed consent was given by interview participants prior to interviews being conducted, with the names of individuals kept confidential and replaced with pseudonyms in all reporting. While no commercial-in-confidence information was sought, and sensitive information was omitted from final publications, permission to identify specific companies was expressly sought and granted by all interviewees. Where appropriate, secondary document analysis – corporate reports, selected media articles and Australian senate submissions – enable us to analyse their narratives, particularly where representatives of agribusiness did not participate in interviews (as was the case for Wilmar and COFCO). The paper also draws on a subset of literature, data and findings generated in a four-year Australian Research Council project [see Ethical Issues]exploring the financialisation of agriculture and farmland in Australia, including 98 interviews in total. The themes arising from this larger pool of interviews have been informed our contextual understanding of the actors, patterns, drivers and tensions associated with financialisation in Australia more widely; this paper draws only on interviews that directly speak to the case study’s focus on sugar investment.

Data were analysed following a two-tiered process of deductive and inductive coding,^47^ which was applied to the entire dataset of 98 interviews. This enabled the identification of thematic categories from both existing theoretical ideas about financialisation (such as the importance of speculative versus productive drivers; roles of different types of financial investors; attitudes towards foreign investment; processes supporting farmland ‘assetisation;’ understandings of responsible financial investment) as well as from the raw data (including the ways finance actors aim to measure social or environmental performance; finance’s role in supply chain consolidation; farmland – values, tenure, land use change; changing investment models; the role of the state; outcomes – food security, financial growth etc; and perceptions around SLO). Each theme included multiple sub-themes (or nodes, derived from axial coding), with coding conducted by multiple researchers (the authors) to improve the reliability of the analysis. Together we identified some 20+ key themes across the entire dataset, with five pertaining specifically to financial investment in the Nth Qld sugar industry. These were:

1. Growth in farmland investment by foreign agribusiness, driven both by demand for processed food inputs and for new financial products.
2. Capital accumulation strategies relating to supply chain consolidation, financial risk mitigation and corporate expansion, including ‘flexcrops.’
3. Food and energy security as ‘legitimate’ drivers for agribusiness investment.
4. Public perceptions of risk (social and environmental) and trust associated with the sugar industry in Australia.
5. Interest in, and compliance with, corporate ESG criteria, ‘ethical investment’ and SLO ideas.

The remainder of this paper seeks to advance understandings of financialisation in Australia by considering how these four companies’ efforts to establish or maintain a SLO (ie, legitimacy, credibility and trust) intersect with their capital accumulation strategies of supply chain consolidation and ‘flexing’ (ie, biofuels production). We apply Borras and colleagues’ concept of flex narratives^1^ – the ‘storylines’ agribusinesses employ when jumping between justifications for capital accumulation strategies – with added emphasis on the limits of these in relation to the health dimensions of sugar. Below, findings are organized by two ‘narratives’ that emerged from interviews: constructing trust and credibility through compliant supply chains; and, biofuels expansion as a legitimate response to climate change. The paper concludes by returning to the concept of social license and its limits regarding health and finance.

## Results

###  Narrative 1: Compliance Across Supply Chains Defines Responsible Investment 

Public perceptions about the health effects of sugar have strongly affected the industry in Queensland. While global per capita sugar consumption is expected to remain stable in 2020, in the medium term it is expected to decline as a result of changing consumer diets and greater concern over health issues.^48^ A recent consumer survey by the Australian Sugar Milling Council^49^– prompted by a “dramatic drop in public confidence” (p. 1) due to increased awareness of wellness and obesity as key elements of a national public health challenge – reported that 49% of respondents were negative or very negative about sugar and health. Specifically, respondents were most concerned about the impact of sugar consumption for high-risk groups (overweight, high consumers of sugar, people with diabetes), the amount of sugar in foods, and the quality of sugar information on food labelling. Women were more likely to be critical or ‘very negative’ about sugar and health. Research has also found that there is growing public awareness of the issue and strong public support for policy interventions in this space.^50^ Despite this, the Australian government has rejected calls from the health industry to implement a levy on sugary drinks, with which there is a direct relationship with long term weight gain and risk of type 2 diabetes.^51,52^ This contradicts recommendations of the 2018 Parliamentary enquiry that supported a 20% tax on sugary drinks, major changes to advertising unhealthy food to children, and less intervention in food labelling and policy-making by the food processing industry.^53^ In short, public trust in both government and industry to address the connection between sugar consumption, obesity, and health is low, of which the sugar industry is acutely aware.

As financial entities whose primary concern is to generate profit for investors, agribusinesses engage with public health concerns quite differently, however. Although “investors are very, very [concerned about] their corporate governance” (Interview 1, agribusiness manager), interviewees were also aware that ESG for financial investments in agricultural supply chains (including land) is in its infancy. This is despite decades of CSA practices and regulations becoming a standard element in agribusiness’ risk management and public relations practices. Ethical investment in agriculture was described by reference to “avoiding the things which are more objectionable to our clients, and I guess by extension, civil society” (Interview 2, investment firm intermediary). They use exclusion policies to avoid socially ‘risky’ investments and emphasise ESG where and when it affects returns on investment.^54^ In the agricultural space, they extend caution around animal welfare and live exports, soil degradation, child labour, worker safety and risk management, all of which were described as similar to increasing regulation in the garment industry and mining or to the rejection of investment in arms manufacturing and tobacco.

 None of the financial actors we interviewed described agribusiness’ social license in terms of the impact of sugar production or consumption on human health. Rather, interviewees were concerned that sugar has not been seen as a desirable investment by institutional or agribusiness investors. As a spokesperson of the firm Racecourse Projects explained:

*“I think the sugar industry has been very closed shop. It’s very complex. If you Google the industry, it’s all negative and all you see is court cases and fighting. Institutional investors don’t want anything tainting their image. I don’t see any wanting to [invest] until it’s very clear what the rules are and there’s no confusion or negativity … Anyone from the investor world, they might have some experience in grains or horticulture, but when they start looking at the politics of the sugar industry, the costs start ratcheting up and they run away”* (Interview 3, Racecourse).

Racecourse Projects is a joint venture (JV) between Proterra Investment Partners^[4]^ and Mackay Sugar (a local sugar milling business), which was looking for investors specifically willing to expand land under sugarcane cultivation. The resulting partnership enables Racecourse to focus on purchasing “either a going concern sugarcane farm or a [beef] farm that has been developed into a sugarcane farm” (Interview 3, Racecourse). The JV means that Racecourse follows two capital accumulation strategies. First, they aim to increase production by horizontally expanding their land ownership ‘footprint.’ Second, they can expand vertically by owning some land, some refineries and some marketing, thus also consolidating their ownership along the supply chain. Capital growth is increasing via Racecourse Projects’ land ownership, while Proterra expands its financial portfolio by purchasing small parcels of land through subsidiaries in strategic growing regions.

Wilmar Australia – a wholly-owned subsidiary of Wilmar International, Asia’s leading agribusiness group – has also pursued strong supply chain consolidation in Australia. Wilmar Australia owns and farms around 6600 ha of agricultural land in North Queensland and leases an additional 2000 ha to local sugarcane farmers; it also own 8 sugar mills along with rail infrastructure. In 2011, Wilmar acquired Australia’s largest raw sugar exporter, Sucrogen (formerly CSR Sugar) and later Proserpine Sugar. This makes the company Australia’s largest producer and exporter of raw sugar; largest renewable energy generator from cane biomass; second largest producer of fuel ethanol; largest sugar refiner (though JV with Mackay Sugar); and, leading exporter to Asia through strategic interests in sugar marketer Queensland Sugar Limited.^55^

 The shared perception across our case studies is that the sugar industry is not well understood by either the public or investors, which is driving industry-wide awareness of their collective need to meet established sustainability standards. As a spokesperson from Racecourse remarked, “they want [their investment] to be legal … they will ask how much it will cost to fix it - if the gap is too big it affects the return on investment and they won’t invest” (Interview 3). Fear of losing international markets, and therefore failing to attract institutional investment, is a strong motivational factor to comply with voluntary standards. As one interviewee explained:

*“99% of all institutional investors today are signatories to the UN PRI and they expect that any investment they make into any company or any industry complies with these ESG protocols … unless the industry as a whole is compliant, they won’t invest*” (Interview 4, agricultural fund manager).

Here, we see the first social license narrative emerging: capital accumulation by supply chain consolidation is both socially and financially desirable, as it helps to mitigate financial and reputational risk for investors. Responsible investment standards play their role to the extent that they reduce agribusiness’ risk at as many points in the supply chain as possible. By connecting supply chain consolidation with mitigating financial ‘risk,’ agribusinesses are consolidating their legitimacy as corporate providers of food security. In Racecourse’s strategy of consolidation, responsible investment was seen (by them) to rest upon the adherence of their financier, Proterra, to established legal requirements and labour rules. Proterra is signatory to the Principles of Responsible Investment (PRI), and they emphasise labour standards, environment (including pollution and climate change mitigation) and good governance. Their ESG statement also aims to “minimize negative social and economic impacts of land acquisitions and agribusiness operations.”^56^ This perspective reflects the strong discourse of private, voluntary standards in industry documentation^57^ as well. It also reflects the belief of many agribusinesses “that the world is running out of food” and that we “need more food to feed the world” (Interview 5, Proterra). Neither challenge the objectives of financial capital in which “pushing unhealthy but profitable food”^23^ (p. 761) remains perfectly acceptable.

By contrast, Wilmar is not a signatory to the PRI or any other international responsible investment standard, and they make no mention of standards specific to sugar^[5]^. Although their international website states that “certification is an important aspect in Wilmar’s sustainability journey,”^58^ the accountability of their investments (as distinct from sustainability) is extremely difficult to ascertain, considering that Wilmar’s financiers have not been able to ensure, enforce, implement or monitor their own ESG policies across Wilmar’s global supply chains. Wilmar has a long track record of negative environmental and human rights impacts associated with land grabs, mostly related to its palm oil operations in Nigeria, Uganda, Liberia, Indonesia and Malaysia.^59,60^ They have also been criticised for their links with Archer Daniels Midland, which has been described by GRAIN^61^ as the “worlds’ worst environmental offender” due to both companies’ use of offshore tax havens, transfer pricing schemes, and illegal environmental activities. Since 2010, Wilmar’s entry into the Australian sugar industry has been marred by disputes over sugar marketing between the company, farmers and the wider industry.^62^This has been detrimental to their SLO in North Queensland, with the Australian Competition and Consumer Commission receiving over 2600 objections from farmers during a 2014 inquiry. Canegrowers media releases from around that time indicate a loss of legitimacy, transparency and trust in Wilmar:

*“If Wilmar was genuinely listening to grower feedback … they would have corrected their unpopular plan to strip away growers’ only trusted marketing and pricing body which gives them unrivalled transparency. Our growers are rising up … they will not have their rights stripped away – even by a large corporate entity such as Wilmar.”* ^63^

*“Wilmar has finally come out and shown its true colours with a blatant statement in a press release that ‘growers do not have any legal or contractual rights.’”* ^64^

*“At a time when industry should be coming together to agree a mutually beneficial and financially sustainable future … growers are right to question Wilmar’s motives.”* ^65^

 From our interviews, it appears that using a narrative of compliance with responsible investment principles – as Racecourse has done, but which Wilmar has not – is a minimum requirement for establishing a social licence to operate, leaving companies free to pursue supply chain consolidation under the banner of providing food security. Questions about the quality and health of diets are missing within this narrative, however, making it possible for agribusinesses to detract attraction from other potentially negative impacts of supply chain consolidation, such as the health impacts of sugar consumption.

###  Narrative 2: Investing in Bioenergy Is Responsible Investment 

In 2011, Mitr Phol purchased MSF Sugar – Australia’s largest sugarcane farm owner, second largest raw sugar exporter, and third largest miller. Based in Thailand, Mitr Phol is the world’s fifth largest sugar producer globally, and the largest in Asia, with aspirations to be “kitchen of the world.”^66^ Prior to its acquisition, MSF had been gradually purchasing land and mills to build the company to a scale that would attract institutional investment. This consolidation of assets was achieved via JVs with local and foreign institutional investors, which facilitated a stepped approach to supply chain consolidation, as well as a way to mitigate financial risk. Mitr Phol now own over 10000 hectares of sugarcane farmland in three different locations in Australia, as well as mills, each one of which is an ‘investor-ready parcel’ large enough to be bought out by an institutional investor. They are also the major investor (19%) in port infrastructure, Sugar Terminals Ltd, indicating a strategic, financially-motivated, supply chain acquisition that allows them to “have financial skin in the game” (Interview 6, Mitr Phol). As a representative explained:

*“We went into a joint venture with all the mills up in the north and we had an option to buy them out. This gave us the ability to bring everything together, run it effectively because we were a JV partner. But having the option to buy it out at a fixed price then gave us time to go to market to get the capital and then buy it out. So, it was all about being able to grow the company and then be able to market the story out to investors. That’s what we did” *(Interview 6, Mitr Phol, emphasis underlined).

This strategy rejects the food security narrative for land purchases,^67^ instead emphasising the benefits of consolidation for expanding their biofuels agenda. The company has around half its assets in processing mills, which they predict to be more profitable in the long term than the land investment. This is based on their capacity to generate electricity and ethanol, in an example of ‘anticipated flexing.’^1^ This involves producing multiple products and services from existing mills, and through a $75 million Green Energy Power Plant investment in 2017, potentially doubling the value of cane. In Thailand, however, Mitr Phol has been the subject of a class action lawsuit for human rights abuses and land grabbing for biofuel plantations in Cambodia. It withdrew from that project in 2014, and the class action was rejected by the Thai civil courts in 2019.^68^

This second accountability narrative is one in which agribusiness frames responsible investment around innovations that increase sugar’s value as a green energy provider in the future. Queensland’s sugar mills have long generated renewable energy from crushing and burning the sugarcane by-product ‘bagasse’ and produce 27% of Queensland’s total renewable energy (over 2% of Australia’s total large-scale renewable energy target).^69^ The risk to investment posed by climate change was widely acknowledged across all our interviews, and emphasising sugar’s positive contribution was explicitly seen to be “part of our SLO” (Interview 7, investment fund manager 1). Companies also wanted to be seen to “ensure we apply our resources and our time to matching that risk” (Interview 8, investment fund manager 2). This is in line with the sugar sector’s plans to lead the development of a ‘biofutures road map.’^70^

Tully Sugar is a wholly-owned subsidiary of COFCO, China’s largest state-owned agribusiness enterprise and the world’s fourth largest agricultural commodity trader.^71^ COFCO International also has close financial ties with Louis Dreyfuss, one of the ABCDs. While not the largest sugarcane landholder or exporter, COFCO owns the largest single processing mill in Australia. This has made them one of the most significant contributors to renewable energy production in the region, generating 99% of their own energy and providing over 10MW/year back to the grid since 1996. This “represents the limit of [their] current generating and transmission capacity”^72^ and has only recently been eclipsed by the expansion of the Mitr Phol mill.

COFCO’s purchase of Tully Sugar in 2013 was met with substantial public criticism on the basis of concerns about foreign investment from the Chinese government^[6]^. In the seven years since, however, COFCO has used its biofuel capacity to improve public support. Their legitimacy is framed around the promise of future innovations the company can offer, in a context of both climate and financial uncertainty. COFCO’s reporting on its global sugar operations emphasises ‘responsible sugar cane cultivation,’ ‘improved climate resilience,’ ‘reducing the carbon footprint,’ ‘divestment from non-renewables’ and ‘closing the loop.’ Their sustainability strategy is the only one (of the four cases discussed here) aligned with the UN Sustainable Development Goals.^73^

 Both Mitr Phol and COFCO are building legitimacy and trust in ways that are more in line with SLO than with voluntary accountability standards. There is, as yet, little public debate around ‘flexing’ in Australia and, in our interviews, few industry experts were familiar with the term. But it has potential to create social contestation. Thus, companies wanted “more control over [their] own destiny” (Interview 6, Mitr Phol) by strengthening normative links with growers and with end consumers of energy, not just sugar. External (public, investor) perceptions of the future of sugar were strong drivers for reimagining the industry, as described below:

*“We’ve got to change it from being a sugar industry to a sugar cane industry. A biomass industry. We’ve got to maximise the products. We’ve got to change the external industries’ perception of what we do. I mean at the moment if you went around here and asked people about sugar, there’s two things they’d probably tell you – One is we make people fat, and two is we’re destroying the reef. People have got to see us as being part of the future …We can make fuel for cars. We can make a whole variety of different foods, not just sugar. So, that’s our challenge. By doing that we increase the revenue for the area” *(Interview 6, Mitr Phol).

 By shifting the public gaze to the positive benefits of the sugar industry for clean energy generation, the negative health impacts of its consumption are again avoided. By extension, agribusiness’ power to define the terms of future fuel investment will likely be a key determinant of how the industry’s SLO might evolve in future.

###  Discussion: Where Is Health in Agribusiness’ SLO Narratives?

In Australia, there is a growing emphasis – from within the industry, and from the public – on the need for financial actors to demonstrate sustainability, responsibility and accountability in their agricultural investments. The concept of ‘SLO’ is increasingly invoked by agribusiness to indicate that their activities are legitimate in the eyes of society, or to overcome possible social disapproval, regardless of whether societal expectations are embodied in law.^74^ We have argued that public health is not widely considered in agribusiness investment decisions, nor is it part of SLO narratives, despite growing critiques of the health implications of processed foods within an increasingly financialised food system.

In the cases of Wilmar, Mitr Phol, COFCO and Proterra, two main narratives are used by these agribusinesses as they seek to establish (or repair) trust, credibility and legitimacy within the sugar industry. Both narratives emphasise the logics of supply chain consolidation and biofuels investment by financiers in order to ‘solve’ the risks to capital posed by global food and nutrition insecurity and climate change, at the same time as generating high profits. In the first narrative, the measure of meeting a SLO is based on compliance with established accountability criteria along the whole sugar supply chain. Both Proterra and Wilmar seek legitimacy for capital accumulation through the diversification of assets (land, crop types, regions) via vertical and horizontal expansion. In turn, this also extends the narrative of food ‘security’ that is widely used by investors to reinforce their role as legitimate actors in ensuring sustainable food markets. Meeting environmental and labour standards were a given, and many of our interviewees were also sensitive to emerging consumer food ethics (eg, animal welfare), in line with established literature on CSR.^75,76^ However, only Proterra was a signatory to the investment-specific *Farmland Principles* (industry-led PRI), despite this failing to “create a strong framework to address the sustainability challenges of biofuels”(p. 151).^37^ Proterra and Wilmar want to be evaluated on the supply chain standards they subscribe to, so as to ‘earn’ a social license to pursue increased consolidation of their financial (and productive) assets. Transforming voluntary standards (including those specific to investment) to include a stronger focus on public health might go some way towards bringing health outcomes into the evaluation of investment risk; although the well-documented limitations of corporate, voluntary standards’ effectiveness would likely remain.^77^ Critics – including local farmers and the public – point out that their performance does not always match their rhetoric.

The second narrative stems from the optimistic portrayal of investing in biofuels as a response to the ‘ethical’ issue of climate change. This was demonstrated by Mitr Phol and COFCO, both of which emphasised the benefits of sugar’s flexible-ness and multiple-ness in meeting future demand for renewable energy. This obscures the fact that JVs with government and other agribusinesses were purely financially motivated in an attempt to attract large-scale corporate investment into energy production. ‘Flexing’ between sugar and biofuels is primarily a financial strategy – aiming for maximum profit, even when social/ethical considerations might play a role in decision-making. The concept of SLO is much stronger in this storyline, as it extends beyond compliance to include reference to future obligations, visions and broader community goodwill. Both Mitr Phol and COFCO are attempting to mitigate negative social reputations though a biofuels *strategy* that aligns corporate and community interests to minimize business risk and a *moral framework* that aims to “meet the often higher set of standards related to social obligations towards local and affected communities” (p. 2).^78^ As long as the moral obligations of agribusiness financiers to transform food systems in a direction *away* from climate disaster and food insecurity remain ill-defined and un-related to shareholder profit, it is unlikely that SLO alone will be enough of a motivation for sector-wide change.

Each of the cases discussed here has met with very different public perceptions of their impacts and responsibilities for the sector and surrounding communities and environments; they are *all *strongly aligned with the growth of biofuels and unhealthy, processed, foods; and, they *all *have historical records of serious legal and environmental breaches in other countries. However, it is extremely difficult to identify who specifically is responsible for the negative consequences of financialised, agribusiness-dominated food chains. For sugar in particular, McKay et al^79^ argue that flexing involves increased horizontal and vertical linkages between sectors, actors, value chains, technologies and discourses that have “led to increased monopolization and concentration of capital” (p. 198). This intensification highlights the almost impossible task of disentangling ownership structures. This is certainly true of finance in the North Queensland sugar industry, as the large agribusiness investors further consolidate their financial power through strong ties to the ABCDs^[7]^.

Transparency in financial investments is obscured by the complex ‘web of institutional power’^8^ created by the diversity and complexity of investor relationships across multiple countries and at different points of the investment chain. Clapp^21^ attributes this to ‘distancing’ associated with financialisation, whereby distance (physical and cultural) between farm and plate, the abstraction of physical commodities into market derivatives, and the subsequent lack of transparency “creates space for competing narratives – often advanced by the financial actors themselves – that point to other explanations for negative social and environmental outcomes” (p. 2). Distancing also means that farmers have less of a stake in productivity and sustainability practices, civil society activists do not know who is responsible, and key decision-makers can remove themselves from the social and ecological consequences of their decisions.^80^ In this study, narratives about strengthening corporate sugar supply chains in order to address food security and climate change deflects the public’s attention from other potential narratives about sugar and public health. Still, alternative narratives do exist – such as those coming from public health on ‘sustainable diets,’ or even from agri-food scholars in the wake of COVID-19 – indicating potential spaces through which to expand public expectations of agribusiness and finance.

## Conclusion

Who benefits and who is disadvantaged from what is said and not said in the construction of social license narratives? In bringing together the concepts of flex-narratives and SLO, the case studies in this paper have shown that the complex interplay between health impacts of a highly corporatised sugar industry are largely considered to lie outside the social purview of financiers. This ‘distancing’ between responsible ‘intentions’ and real-world outcomes raises ongoing questions about the legitimacy of finance capital to determine the appropriate health, environment or food security outcomes for society.^84^ Sustainability reporting, for example, is not equipped to measure corporations’ contributions to deeper structural changes, such as the concentration of power, privilege and influence in global supply chains.^85^ By contrast, SLO’s responsiveness to the changing boundaries of social legitimation differentiates it from other vehicles that have been co-opted by industry (such as CSR and CSA), and represents an important – although under-researched – opportunity for the longer-term transformation of food systems.

This paper has illustrated the ways that social license narratives within the sugar industry in Australia emphasise (*a*) compliance with voluntary *principles of responsible investment*, (*b*) building a business case for sustainable energy supply chains, and (*c*) a combination of both. The health implications of sugar consumption by distant consumers in global supply chains does not enter financiers’ calculations of business risk, and public health concerns do not appear to have influenced sugar financiers’ storylines as they seek to legitimise their capital accumulation strategies and build public trust. Within a financialised food system, expanding supply chain accountability to include the public health implications of sugar consumption would be akin to the tobacco industry supporting restrictions on smoking; a demand fraught by political power and financial influence. This highlights the limits to principles of responsible financial investment and questions the legitimacy of finance to claim a SLO.

## Acknowledgements

 Dr. Kiah Smith is funded by the Australian Research Council (DE190101126). Emeritus Professor Lawrence is grateful for funding from: the Australian Research Council (DP 160101318); the Ministry of Education of the Republic of Korea and the National Research Foundation of Korea (NRF-2016S1A3A2924243); and, the Norwegian Research Council (FORFOOD No. 220691).

## Ethical issues

 This study adheres to the Guidelines of the ethical review process of The University of Queensland and the NHMRC guidelines for the ethical conduct of research; Approval number 2016000410, ‘Food, farming and financialisation: Agri-food transformation in Australia’ (June 2016 to December 2021).

## Competing interests

 Authors declare that they have no competing interests.

## Authors’ contributions

 KS: 80% - research design, data collection, data analysis, writing. GL: 20% - research design, writing.

## Funding

 Australian Research Council Discovery Project No. DP 160101318, ‘Food, Farming and Financialisation: Agri-food Transformations in Australia’, G. Lawrence, Principal Investigator, June 2016 to December 2021.

## Endnotes


^[1]^ The four largest global agri-food commodity traders are Archer Daniels Midland, Bunge, Cargill and Louis Dreyfus – the ‘ABCDs.’ They play a crucial role in the operation of food, feed and fuel complexes and use their power to profit from the sale of grains, seeds and agro-chemicals. Despite being implicated in food price volatility, many of their dealings are not disclosed to the public.^13^

^[2]^ Bjorkhaug et al^11^ describe the key lines of enquiry to include global food regime dynamics, such as farmland ownership, commodity trading and retailing; conceptual connection to globalisation, corporatisation and neoliberalism; connection of food price volatility and commodity index speculation to food (in)security; documenting emerging financial models, motives and actors changing investment relationships; farmland ‘assetization’ processes; the role of the state and civil society; critiques of impacts; and resistance.

^[3]^ It is beyond the scope of this paper to investigate this emergent literature, which includes special issues on ‘Agriculture, Food and Covid-19’ (*Agriculture and Human* Values 37, 2020) and ‘COVID-19 and the Canadian agriculture and food sector: Thoughts from the pandemic onset’ (*Canadian Journal of Agricultural Economics* 68/2, 2020).

^[4]^ Previously, Black River Asset Management.

^[5]^According to their website, Wilmar International is a member of the Roundtable on Responsible Palm Oil, Global Compact, International Sustainability & Carbon Certification (covering bio-based feedstocks and renewables), and have their own No Deforestation, No Peat, No Exploitation policy.

^[6]^ The negative attitudes towards investment from China has been examined by financialisation scholars elsewhere.^81-83^

^[7]^ The only ABCD not represented here is Bunge – they led an unsuccessful bid to purchase Tully Sugar in 2012.

